# Medical and Behavioral Aspects of Adolescent Endometriosis: A Review of the Literature

**DOI:** 10.3390/children9030384

**Published:** 2022-03-09

**Authors:** Maria-Konstantina Liakopoulou, Ermioni Tsarna, Anna Eleftheriades, Angeliki Arapaki, Konstantina Toutoudaki, Panagiotis Christopoulos

**Affiliations:** Second Department of Obstetrics and Gynecology, “Aretaieion” Hospital, Faculty of Medicine, National and Kapodistrian University of Athens, 11528 Athens, Greece; m.liakopoulou@med.uoa.gr (M.-K.L.); ermina1990@windowslive.com (E.T.); melefth@med.uoa.gr (A.E.); aarapaki@gmail.com (A.A.); konstantinatoutoudaki@gmail.com (K.T.)

**Keywords:** endometriosis, adolescent, dysmenorrhea, stress

## Abstract

The majority of young women will experience discomfort associated with menstrual cycles and miss out on education and social opportunities. Endometriosis, the presence of endometrial glands and stroma outside of uterus, is the most common cause of secondary dysmenorrhea and characterized by pain despite treatment with nonsteroidal anti-inflammatory drugs and hormonal agents. The true prevalence of adolescent endometriosis is not clear. Delay in diagnosis leads to persistent pain, affects quality of life, and potentially contributes to disease progression and subfertility. A laparoscopic diagnosis is the gold standard, but the surgical appearance may differ from adults, as endometriotic lesions are usually red or clear, making their identification a challenge for gynecologists who are unexperienced with endometriosis in adolescents. A personalized medical–surgical treatment is regarded as the most effective therapeutic strategy to achieve remission of symptoms, suppress disease progression, and protect future fertility. Studies have demonstrated how adolescent endometriosis negatively affects patients’ quality of life and psychosocial functioning. Development of therapeutic interventions targeting psychosocial function and quality of life is imperative for adolescent patients.

## 1. Introduction

The majority of adolescents (70–93%) experience discomfort associated with menstruation [1,2]. Dysmenorrhea is the most prevalent gynecologic complaint in adolescents and young adults and one of the most frequent causes of educational and occupational absenteeism [3]. Furthermore, adolescents with severe dysmenorrhea are at greater risk for anxiety and depression [4].

Most adolescents have primary dysmenorrhea, defined as painful menstruation, in cases that pelvic pathology has been excluded [5]. The pain and discomfort emerge when ovulatory cycles are attained, usually within 6 to 12 months post menarche. These symptoms may begin the last day of the menstrual cycle, continue during the first two days of menstruation, and be accompanied by nausea, vomiting, muscle cramps, and headaches [6,7]. The pathophysiology of primary dysmenorrhea includes increased uterine contractility and elevated levels of prostaglandins, as the ischemic endometrium is shed [1]. A two-fold increase of PGF2a activity level has been found in dysmenorrheic women’s menstrual fluids, compared to eumenorrheic women [8]. Additionally, levels of leukotrienes are higher in the urine of dysmenorrheic women [9]. Prostaglandins and leukotrienes are both inflammatory mediators and, in combination with vasoconstriction, are considered as the main cause of pain.

Secondary dysmenorrhea refers to painful menstruation, which is attributed to varying underlying gynecologic or other medical conditions. The condition usually appears a few years after menarche, and pain usually worsens over time. Failure to respond to first line treatments is more common among patients with secondary, rather than primary, dysmenorrhea [3,6,10]. Among the potential causes of secondary dysmenorrhea, endometriosis is more commonly implicated [11,12]. Other probable causes are adenomyosis, genital tract infections, fibroids, Müllerian anomalies, obstructive reproductive tract anomalies, uterine polyps, ovarian cysts, and pelvic adhesions [6].

Endometriosis is a gynecologic disorder, defined by the presence of endometrial glands and stroma outside of the uterine lining. It is characterized mainly by pain and infertility. Endometriotic implants are usually discovered within the female pelvis, most commonly on the ovarian surface and peritoneum. They are more commonly located in the left pelvis, possibly because rectosigmoid acts as a shelter to the cells during the clockwise peritoneal current [13]. Interestingly, implants in distant locations, such as the upper abdomen or the lungs, are also described [14,15]. Due to limited epidemiological data, it is difficult to establish a consensus approach for adolescent endometriosis.

## 2. Endometriosis Epidemiology and Risk Factors

The exact prevalence of endometriosis among adolescents is yet to be estimated, as the reported prevalence varies among studies, depending on the population examined. In particular, 73% of patients, both adolescents and adults, with a history of severe primary dysmenorrhea, as well as between 19–73% of women undergoing diagnostic laparoscopy for chronic pelvic pain (CPP), are diagnosed with endometriosis [16,17]. A systematic review, published in 2013, concluded that endometriosis is present in 62% of adolescents that undergo laparoscopy [12,18]. In detail, endometriosis was laparoscopically confirmed in 75% of adolescents with treatment resistant CPP, 70% of adolescents with dysmenorrhea, and 49% of adolescents with CPP [12].

Assessing and diagnosing endometriosis in adolescents is challenging. Dysmenorrhea, which characterizes endometriosis, is a quite common symptom in adolescents, affecting 40–50% of them. Even though diagnostic laparoscopy is needed for a definite diagnosis, it is rarely performed in adolescents, since watchful waiting is usually preferred. Moreover, indications for laparoscopy are not clear. For instance, CPP is defined differently in individual studies; as a result, patients who undergo diagnostic laparoscopy are usually those with more severe symptoms. Additionally, adolescent girls with endometriosis might be asymptomatic or have atypical presentation. Endometriosis may be a progressive disease, demonstrating its first clinical manifestations several years after menarche. Nonetheless, two-thirds of women with endometriosis report onset of symptoms before turning 20 years old, occasionally as soon as one month after menarche, and even before menarche, in the absence of obstructive anomalies [19,20].

Among the most important risk factors for endometriosis in adolescence are the young age at menarche, co-presence of obstructive Müllerian anomalies, and positive family history. Several twin studies indicate the influence of environmental parameters, which interact with genetic factors, ultimately increasing the individual risk for endometriosis [21,22,23]. In a case-control study that included 123 patients with histologically confirmed endometriosis, the condition was also diagnosed in 6.9% of their first-degree relatives, compared to 1% of controls’ relatives [24]. This finding supports the hypothesis that endometriosis is a multifactorial disorder characterized by genetic predisposition. Women with early menarche have greater cumulative exposure to menstruation, as is the case for women with shorter cycle length, longer duration of menses, and/ or increased menstrual flow. Similarly, higher levels of endogenous estrogens seem to increase the risk for endometriosis, as obese adolescents face greater risk [10,25,26,27]. Obstructive Müllerian anomalies, such as imperforate hymen, transverse vaginal septum, and vaginal agenesis, are also considered important predisposing factors, due to retrograde menstruation [28,29]. In addition, sociodemographic characteristics have been recognized as predisposing factors; in particular, diagnosis of endometriosis is more likely in Caucasian and Asian women, compared to Hispanic and black women [30]. Finally, early stressful events, such as childhood sexual and physical abuse, have been associated with endometriosis, implicating the hypothalamic pituitary adrenal axis in endometriosis pathogenesis [31].

## 3. Endometriosis Pathogenesis

The etiology and pathophysiology of endometriosis is yet to be explored. Six main theories have been proposed: retrograde menstruation or Sampson’s theory, coelomic metaplasia, hematogenous spread, lymphatic spread, neonatal uterine bleeding, and the immunologic theory.

Sampson’s theory remains the principal theory. During menstruation, menstrual fluid flows backwards through the fallopian tubes. In this fluid, endometrial mesenchymal stem cells, epithelial progenitor cells, and stromal fibroblasts are present and can attach to the peritoneum [32]. In line with Sampson’s theory, due to gravity and the clockwise circulation of peritoneal fluid, endometrial implants are more common in the posterior cul-de-sac, left pelvis, and right diaphragm [33]. However, as interesting as Sampson’s theory is, it cannot explain why endometriosis is present in only 5–10% of adult women, while 76–90% of them experience retrograde menstruation [34].

In coelomic metaplasia theory, the peritoneal coelomic mesothelial cells undergo metaplasia and transform into endometrial cells [34,35]. Therefore, according to this theory, endometrial glands and stroma outside of the uterus do not represent implants.

The aforementioned theories cannot explain how endometrial implants arise outside of the peritoneal cavity. To address this issue, the hypotheses of hematogenous and lymphatic spread have been proposed [36].

To explain the presence of advanced endometriosis in adolescence and endometriosis before menarche, the phenomenon of neonatal uterine bleeding (NUB) has been implicated. Even though only 5% of newborn girls get diagnosed with NUB, 25% of them may experience occult bleeding [32]. Until recently, NUB was considered an insignificant event, but recent studies suggest its importance in the pathophysiology of endometriosis in premenarchal girls [32,37]. NUB is an endometrial response to withdrawal of progesterone. Two-thirds of neonates have endometrium resistance to progesterone, which persists until menarche and early adolescence. NUB has also been associated with fetal distress, due to fetal growth restriction, preeclampsia, postmaturity, and Rhesus isoimmunization. Under the aforementioned conditions, insufficient blood is supplied to the placenta, the fetus may be hypoxic, and decidualization of fetal endometrium and sensitization to progesterone are promoted [38]. During puberty, when the estrogen production increases, the endometrial progenitor cells are re-activated, promoting angiogenesis at the endometriotic implants, which ultimately leads to recurrent implants’ bleeding and endometriomas formation [15,39].

The immunologic theory has been proposed, in order to explain why endometriotic cells can survive and invade tissues outside of the uterine lining, and even induce angiogenesis. In this process, the evasion of immune surveillance, the activation of proinflammatory pathways and the angiogenesis via multiple cytokines have been implicated. Proinflammatory signaling plays an essential role with elevated cyclooxygenase (COX)-2, tumor necrosis factor a (TNFa), prostaglandin E2, growth factors, cytokines, and angiogenic factors [40]. Increased interleukin (IL)-1a, IL-6, and IL-8 regulate cell growth and angiogenesis, and fibronectin promotes ectopic cells attachment. Elevated levels of vascular endothelial growth factor (VEGF), macrophage-derived growth factor, and monocyte chemotactic protein (MCP-1) enhance the proliferation of fibroblasts and endometrial cells [41]. Changes in levels of complement proteins are also observed in patients with endometriosis. Latest studies suggest the importance of the complement system within the pathophysiology of endometriosis and underline the role of C6, especially during early disease-stage [42].

## 4. Clinical Presentation of Endometriosis

Clinical presentation of endometriosis in adolescence includes more atypical symptoms, as well as complaints from systems other than the reproductive, such as the gastrointestinal and urinary systems. Adolescents may not present with the typical symptomatology of endometriosis, namely dysmenorrhea, dyspareunia, dyschezia, and/or infertility. Common symptoms in young women with endometriosis include abdominal discomfort, chronic pelvic pain, low back pain, heavy menstrual bleeding, headaches, dizziness, low energy, and mental disturbances [26,43]. General abdominal symptoms may include nausea, bloating, tenesmus, diarrhea, constipation, painful defecation, and rectal pressure; pain may improve following bowel movements [44]. In a study of adolescents with laparoscopically diagnosed endometriosis, 56% had at least one gastrointestinal symptom preoperatively, and 52% had one or more genitourinary symptoms [45]. The spectrum of genitourinary symptoms includes abnormal urination frequency and urination-associated pain, as well as abnormal uterine bleeding [26,46,47]. It is worth mentioning that patients with endometriosis could present a variety of clinical manifestations from other systems, as an association between endometriosis and other clinical conditions, such as inflammatory bowel disease, rheumatoid arthritis, asthma [48], psoriasis [28], and migraines [49], is commonly reported.

Endometriosis can get diagnosed before menarche, as has been reported in girls with pelvic pain [50]. Severe dysmenorrhea or drug-resistant pain associated with absenteeism from activities should suffice to include endometriosis in the differential diagnosis. However, among patients with endometriosis, there is no association between the severity of pain and severity or location of disease [46].

## 5. Diagnosis of Endometriosis in Adolescence

Several studies have highlighted the significant time interval between onset of endometriosis symptoms and diagnosis that potentially contributes to prolonged suffering, progression of disease, and fertility impairment [51,52]. Adolescent endometriosis is a challenging diagnosis, and the disease can be easily overlooked. Dun et al. reported that teenagers were experiencing pain for at least 22.8 months and had been evaluated by at least three specialists before endometriosis was diagnosed [45]. Therefore, techniques for early, noninvasive diagnosis are needed to reduce the consequent delay in access to treatment. These techniques should, however, be specific enough to correctly diagnose endometriosis, since up to 12% of teenagers report endometriosis related symptoms, as was shown by Zannoni et al. [53].

Further evaluation should be considered when prolonged use of nonsteroidal anti-inflammatory drugs (NSAIDs) is reported by the patient, there are relatives diagnosed with endometriosis (in cases of frequent absenteeism from everyday activities during menstruation), and estroprogestin contraceptives have been prescribed before the age of 18 years for primary dysmenorrhea treatment [54].

The evaluation of an adolescent, in the case that endometriosis is suspected, should start with a comprehensive medical, gynecologic, and family history. Age at menarche, regularity of menstrual cycle, menstruation duration, amount of menstrual bleeding, and time interval between menarche and dysmenorrhea onset should be recorded. A pain diary might be used to record the pain frequency, as well as the character of the pain and potential triggering factors, such as menstruation or bladder/bowel function, mood, diet, medication, and musculoskeletal symptoms [55].

A pelvic examination should be considered to exclude from the differential diagnosis a pelvic mass or a reproductive tract anomaly. With appropriate support, many non-sexually active adolescents tolerate a pelvic examination. If not, a rectal abdominal exam may be better tolerated.

The initial imaging technique is most frequently an ultrasound examination, which allows for an extensive exploration of the pelvis and can rule out pelvic masses or structural anomalies [56]. However, the easily visualized endometriomas are not very common during adolescence, compared to adulthood, and stage I or II endometriosis (which is more common among adolescent patients) can rarely be visualized through ultrasound imaging [55]. Thus, an unremarkable ultrasound examination does not exclude endometriosis. MRI demonstrates an important advantage over ultrasound because it allows for a simultaneous, complete imaging of all pelvic compartments and is, therefore, thought to be more sensitive in the diagnosis of endometriosis. Nonetheless, in studies that compared MRI to laparoscopy, MRI was less likely to be used [50]. Compared to laparoscopy, endometriosis findings are less likely to be presented on a MRI examination [57]. Furthermore, MRI has not proved to be as cost-effective as an initial screening tool [58].

No specific blood tests or serum markers to identify endometriosis currently exist. Although serum CA 125 levels can be elevated, the American College of Obstetricians and Gynecologists (ACOG) does not recommend the use of CA 125 for patients’ screening or monitoring of endometriosis treatment [14]. Other laboratory studies, such as complete blood count or erythrocyte sedimentation rate, that reflect on the underlying inflammation process have low specificity for endometriosis. In order to rule out other potential causes, urinalysis or urine culture should be performed. Pregnancy and sexually transmitted diseases (STDs) also need to be excluded.

After the exclusion of all other potential causes, NSAIDS and estrogen/progestin or progestin therapy may be chosen. Continuous hormonal therapy can be used to suppress menstruation and is considered safe. With regard to the empiric use of gonadotropin releasing hormone (GnRH) agonist or antagonist, it is not recommended in adolescents with chronic pelvic pain and suspected endometriosis, due to potential impact on bone density, in contrast to adulthood [59]. Menstrual suppression may decrease the need for surgical treatment, due to symptoms remission. However, it cannot be used to treat adhesive disease.

ACOG recommends laparoscopy for diagnosing endometriosis in adolescents [60]. Diagnostic laparoscopy is indicated if there is no relief after 3–6 months of medical management. On the contrary, laparoscopy, without a trial of medical treatment, performed exclusively for diagnostic purposes, should be avoided [50]. However, the gold standard for diagnosing endometriosis remains the histological examination of suspicious lesions, because of the high false positive rate of visual identification during laparoscopy [50]. The benefits of laparoscopy do not only include the confirmation of diagnosis, but also the opportunity of intraoperative treatment.

During laparoscopy, endometriosis may have a variable appearance, as described in the revised American Society of Reproductive Medicine (ASRM) classification of endometriosis [61]. White, yellow-brown, red-pink lesions, as well as clear shiny vesicular lesions, are more frequent among adolescents and associated with greater levels of pain [46]. Due to their subtle presentation, different techniques for lesions’ visualization have been proposed. One of these is the “close tip technique”, according to which the laparoscope is moved within millimeters of the peritoneum; subsequently, the magnification is properly adjusted [57]. According to another technique, the laparoscope should be submerged within the irrigation fluid (e.g., normal saline or lactated Ringer’s), which has been used to fill the pelvis with, before visualizing these clear shiny lesions [57]. Peritoneal Alan-Masters windows are also common in adolescents and indicative of endometriosis [61]. On the contrary, powder-burn lesions are not very common in adolescents, as they correspond to older and more advanced implants. If suspicious lesions are not identified during laparoscopy, random biopsies of the cul-de-sac should be obtained, with the aim to identify microscopic disease [62,63]. ACOG does not recommend “peritoneal stripping” in adolescents because of potential adhesion formation that might contribute to persistent abdominal and pelvic pain, infertility, and even bowel obstruction. In addition, the efficacy and effectiveness of “peritoneal stripping” is still debated [60].

Endometriosis staging should be assessed as described in the revised ASRM classification [61]. Most adolescents present with stage I–II disease; however, advanced stage III–IV disease, including ovarian endometriomas, is increasingly diagnosed in adolescents. The stage and location of the lesions do not directly corelate with the severity or frequency of symptoms. On the contrary, clear and red lesions are associated with greater prostaglandin production, metabolic activity, pain, and inflammation than the usual older powder burn lesions, which explains why patients diagnosed with an earlier stage can suffer from substantial pain [26,45,46,64,65].

Even though laparoscopy has traditionally been considered the gold standard to diagnose endometriosis, it remains an invasive procedure with potential complications. Therefore, robust evidence from large-scale international trials is needed, in order to develop and validate a non-invasive and inexpensive diagnostic tool with high sensitivity and specificity [66].

## 6. Management of Endometriosis in Adolescence and Treatment Options

Endometriosis is a chronic disease and may recur despite therapy. Treatment of adolescent endometriosis has three aims: to control the symptoms, prevent the progression of disease, and preserve fertility. A personalized treatment approach is more likely to achieve the aforementioned goals.

Various studies propose NSAIDs and combined oral contraceptives, in case NSAIDs are not effective, as the first-line treatment [16]. Therapy with estrogen/progestin or progestin alone creates a progestin-dominant environment, making the endometrial tissue atrophic [57]. Progestin-only treatment methods, such as the “mini-pill” (containing norethindrone), induce decidualization, with subsequent atrophy; nevertheless, it has been calculated that 9% of patients will not respond [67]. Depot medroxyprogesterone acetate (DMPA) use is limited, due its association with lower bone mineral density.

Gonadotropin releasing hormone (GnRH) agonist treatment may be considered, if all other possible treatment methods were unsuccessful and only if the patient is more than 17 years old because, at this developmental stage, bone structure is completed [26]. According to ACOG, patients who have pain refractory to conservative surgical therapy and suppressive hormonal therapy advantage from at least 6 months of GnRH agonist therapy with add-back medicine [60]. GnRH agonist use is very effective, for both the resolution of active disease and pain [59]. These agents result in a down-regulation of the hypothalamic-pituitary-gonadal axis, which leads to a hypoestrogenic environment, ultimately suppressing endometriotic lesions. However, the use of GnRH agonists cannot exceed short periods of time, as long-term use may lead to bone density loss and potentially affect negatively cardiovascular risk [67]. Moreover, GnRH agonists, when administered before surgery, change the macroscopic image of endometriotic lesions, make their visualization harder, and, thus, preclude effective surgical treatment. Therefore, GnRH agonists are usually part of the post-operative treatment. In adolescents treated with GnRH agonists, add-back hormone therapy should also be administered, in order to prevent bone density loss and menopausal symptoms [68]. Furthermore, hormonal treatment with add-back therapy is shown to improve bone health and quality of life [16].

As previously mentioned, laparoscopy is the gold standard for diagnosing endometriosis, but also represents a therapeutic option. Gynecologists should be familiar with the appearance of adolescent endometriotic implants, which are usually clear, white, and pink–red and may be different from those in an adult woman. Implants can be destroyed via electrocautery, coagulation, ablation, or excision. The lysis of adhesions should also be performed [69]. A levonorgestrel intrauterine system (LNG-IUS) may be also placed at the time of laparoscopy, as it has been shown to decrease the pain associated with endometriosis [25,66,70]. However, the LNG-IUS is not approved by the U.S. Food and Drug Administration for treatment of endometriosis-associated pain [60]. The appropriate timing of surgery for adolescent endometriosis is still debated. Some specialists recommend avoiding surgery as long as possible, due to high recurrence rates [71]. On the other side, others suggest surgical intervention at the early stage, in order to prevent the development of more severe lesions and eliminate endometriosis completely [71]. Notably, several studies suggested that there is no association of age with progression of endometriosis [40]. On the other hand, other studies suggest that typical lesions of endometriosis can increase over time [33,72]. Therefore, further research is needed, in order to determine the best approach at the proper timing for adolescent endometriosis.

There is controversial data regarding the role of hormonal suppression [46,71]. Currently, the recommended treatment for adolescent endometriosis is combination of surgical and suppressive medical therapies that prevent endometrial proliferation [57]. Since endometriosis constitutes a chronic and progressive disease, it is recommended that patients continue hormonal suppression therapy, unless they wish to become pregnant [60]. Although subfertility is rare in adolescents, in cases of severe endometriosis, cryopreservation of ovarian tissue can also be recommended, due to the possibility of primary ovarian failure and high recurrence rates [40].

Although bigger longitudinal studies are needed to obtain more accurate results, new agents have been suggested as potential treatment options, using animal models. The results of a recent rat study by Hortu et al. indicated that oxytocin and Ankaferd Blood Stopper, a VEGF modulator, could have potential as therapeutic agents in experimentally-induced peritoneal endometriosis [72].

## 7. Social and Psychological Impact of Endometriosis

Endometriosis constitutes a disabling condition, which particularly affects social relationships, as well as sexual and mental health. Several studies have highlighted the impact of chronic pelvic pain on the psychological well-being of women and adolescents with endometriosis, as well as on their life quality. Notably, pelvic pain and infertility are commonly associated with psychic vulnerability and drug abuse. [73].

High levels of anxiety, depression, and other pathological mood symptoms that characterize psychiatric disorders are reported by women who have the diagnosis of endometriosis. Endometriosis can crucially affect the social and psychological domains of women’s lives [74]. In a study by Barnack et al. endometriotic women reported significantly higher stress and anxiety levels and demonstrated pessimistic attitudes towards menstruation, as compared to women with chronic migraine headaches [75]. Petreluzzi et al. showed that women with endometriosis and chronic pelvic pain of moderate intensity had higher levels of perceived stress and a low concentration of salivary cortisol [76]. Beginning from the clinical observation that several women with neuropsychiatric symptoms, seen in psychiatric practice, also had the diagnosis of endometriosis by their gynecologists, Lewis et al. stated the hypothesis that endometriosis could be associated with psychiatric illness. Lewis et al. found a high prevalence of mood disorders in endometriotic patients, suggesting a hormonal mechanism as a potential cause. Nonetheless, his findings were strongly limited [77].

Regarding the psychological impact of endometriosis in adolescents, the available data is also limited. In a study by Rowlands et al., adolescents/young women, aged 18–23 years, who had the diagnosis of endometriosis demonstrated a significant risk of developing moderate to severe psychological distress, in comparison to young women without a history of those conditions. The study by Rowlands et al. included a total of 17,015 young women, born from 1989–1995, who completed a web-based ALSWH (Australian Longitudinal Study on Women’s Health) survey [78]. Gonzales et al. provided additional evidence, demonstrating that endometriosis symptomatology can substantially impair the psychological well-being of young patients. In this study, 24 patients, aged 13–25 years old, completed numerous questionnaires, including the Beck Anxiety Inventory (BAI), Endometriosis Health Patient-5 (EHP-5), and Visual Analogue Scale (VAS) [79].

In addition, in a study by Smorgick et al., adolescents/young women, aged less than 24 years old, had a high prevalence of comorbid pain syndromes, such as chronic headaches, chronic low back pain, vulvodynia, fibromyalgia, and chronic fatigue syndrome. The incidence of asthma and mood conditions, characterized by depressive and anxiety symptoms, was also high. In fact, comorbid pain syndromes were found in 77 (56%) young women, mood conditions in 66 (48%) women, and asthma in 31 (26%) women [80].

As far as dysmenorrhea is concerned, one of the main implications of endometriosis, an observational study by Kabukcu et al. demonstrated that primary dysmenorrhea could also be associated with ADHD symptomatology and psychiatric distress and, thus, affect daily activities. In this study by Kabukcu et al., 104 (49.8%) adolescents stated that the pain that they experienced during menstruation importantly affects their daily activities. These adolescents had diminished sleep quality and more inattention and hyperactivity–impulsivity-related daily life problems. Symptoms of anxiety, depression, negative self-esteem, and hostility were also more frequently reported in the endometriosis group, in comparison to the controls. The severity of menstrual pain, as measured by VAS, was positively correlated with ADHD symptoms, as well as all other psychological parameters [81]. Since primary and secondary dysmenorrhea share, by definition, the same clinical manifestations, endometriosis could be potentially associated with ADHD symptomatology [82].

This evidence suggests that the burden of endometriosis is not limited to the symptoms and dysfunction related to the condition. It affects the social, working, and emotional domains, potentially leading to a severe impairment of global functioning [82]. Endometriosis symptoms, and the related psychological consequences, constitute women more vulnerable and increase the likelihood of psychiatric symptoms, which can influence coping strategies, weakening resilience to daily struggles and triggering a vicious cycle [83]. Robust evidence from further observational studies is necessary, in order to provide a better understanding of the relationship between endometriosis and psychiatric/behavioral disorders. A multidisciplinary approach to adolescents suffering from endometriosis by a medical team, composed of gynecologists, psychologists, psychiatrists, and sexologists, is needed, in order to create an individualized treatment plan [83]. This approach could facilitate the early detection of psychopathological conditions that accompany endometriosis and improve the physical symptoms of this condition, thereby enhancing the life quality of these patients.

## 8. Conclusions

Adolescent endometriosis, a chronic inflammatory disease with social, physical, and emotional consequences constitutes a common condition, whose exact prevalence still remains unknown. Its prompt diagnosis cannot always be achieved, which can lead to suffering for several years. Consequently, early diagnosis appears to be of upmost importance, especially as far as adolescents and young patients are concerned, as it can optimize life quality, relief symptomatology, and decrease the negative impact of the disease on future fertility [82]. A commonly reported approach is a combination of laparoscopy and postoperative hormonal treatment; nonetheless, recurrence remains a significant issue. The need for more prospective studies investigating the long-term benefits of hormonal suppression before and after laparoscopy, utilizing combined oral contraceptives and progestins, is evident. Most importantly, it is high time to sensibilize gynecologists and adolescent care specialists and raise awareness, regarding the psychosocial and behavioral impact of endometriosis, in order to provide a holistic approach to patients.

## Data Availability

Not applicable.
